# Treatment of Severe Facial Injuries

**Published:** 1917-12-08

**Authors:** Kelsey Frey, Percival Cole


### TREATMENT OF SEVERE FACIAL INJURIES.

**By Major Gillies, Captain Kelsey Frey, and Mr. Percival Cole.**

At the ordinary meeting of the Medical Society of London on Monday, December 3, under the presidency of Sir St. Clair Thomson, M.D., a remarkable exposition and demonstration of the treatment of various types of severe injury of the face, including the jaws, inflicted in the war, was given by Major H. D. Gillies, R.A.M.C., Captain Kelsey Frey, and Mr. Percival Cole, aided by the exhibition of numerous patients and a large number of photographs. The very large attendance included Mr. Hodge (Minister of Pensions) and Dr. Lynch, M.P.

Major Gillies said his wish was to bring the resources of science and surgery to the solution of the problems created by the severe facial wounds which so many of the soldiers had received in this war, these problems having been complicated by the necessity to send back to his duty the victim of such disfigurements, because in these circumstances the cosmetic effect must take a secondary place to that of restoration of function. He spoke from the material which 600 cases under his responsibility gave, and believed that the need for the fitting of artificial noses, cheeks, etc., became less as surgery for these cases became better. Some alleged that the treatment of these cases was comparatively easy, as the principles of plastic surgery were already well known; but to that statement he demurred. The first great difficulty was the restoration of the facial contour, and the second was the due rebuilding of the cartilaginous and bony scaffolding. He was now able to feel fairly certain, in a given case, what was the best form of surgery to apply. There was a great temptation to look around for any form of foreign body with which to make a successful facial contour, but it should be resisted.

In pre-war days the disfigurement resulting from removal of the jaw for cancer had been somewhat made good by the use of metal jaws; but his view was that only one method was available which gave promise of good and anything like permanent result, and that was the transplantation from another part of the body of normal, or nearly normal tissue. For rebuilding the destroyed substructure of the nose, cartilage was preeminent, and in many cases it had been found possible to line or rebuild a nose. In some cases a very large lower jaw loss had been to a considerable extent made good where it seemed impossible to expect a bone graft could succeed. There was a large field of usefulness in bone-grafting on the instalment system. The loss of muscle was often a serious drawback to an otherwise successful plastic procedure. Good function and a good appearance generally went hand in hand. He found that in the mouth region a flap which had its raw under surface exposed would produce cicatricial contraction, and subsequent trouble due to this. To supplement carefully designed mucous flaps he had used small skin flaps—the method known as the epithelial inlay. This was of equal use in the orbit, the nose, and the buccal cavities. The skin graft took as a whole, and every part in contact with living tissue lived. A contracted eye-socket would hold an artificial eye.

Some of the worst cases shown were those due to burns from the German *Flammenwerfer*, for here very little soft tissue remained on the facial skeleton, and the cases were pitiable in the extreme. Almost as severe were those of flying-men who had to descend with their craft alight. In these latter there was a remarkable demarcation of burned tissue determined by the position of the facial mask. Major Gillies described each type in informing detail.

Captain Kelsey Frey dealt with the question of the restoration of the function of the mandible in these cases. He stated the following as the points to be aimed at: (1) The production of bony union; (2) good articulation; (3) the restoration of the mouth sufficiently to enable a denture to be worn; (4) giving the patient the ability to open and close the mouth. His experience led him to say that unless bony union was obtained the power of mastication was lost to an extent varying from 10 per cent. to 90 per cent., the degree varying with the position of the fracture, also with the nature of the union. The method employed was largely dependent on the degree of sepsis present, and the amount of bone lost. In the severest cases the plan was to hold the soft and hard parts in good position, and wait until surgical treatment became possible. The methods which had been employed came under one of the following heads:—Freshening in addition to a periosteal graft; wiring, with periosteal graft, bone slide, bone graft. These the author described in detail, and showed examples by means of the epidiascope. In all cases of gunshot wound first consideration was given, by himself and colleagues, to secure good bony union, and, whenever possible, to combine it with good articulation. He spoke highly of the assistance given to the cases in the matter of wiring.

Mr. Percival Cole urged that these cases should receive the combined efforts of the general surgeon and the dental surgeon, the treatment of these injuries being viewed as a whole. To be of service the primary suturing should be done quickly, and in deciding on the course of procedure, and the subsequent manipulations, functional considerations should always be held to take precedence of merely cosmetic ones. In some of the cases the insertion of any form of splint had been found to be impossible without first resorting to surgical measures. In making a flap skin was an admirable substitute for mucous membrane. He had found that irradiations had rendered great assistance to the surgeon, and for this reason he considered that an $x$-ray installation should be part of the equipment of every jaw centre and hospital in the country.

The exhibitors were cordially thanked for the great trouble they had taken and for their desire to let their brethren know what was being done for this pathetic type of case.

